# Editorial: Obesogenic Environmental Conditions Affect Neurodevelopment and Neurodegeneration

**DOI:** 10.3389/fnins.2021.724503

**Published:** 2021-08-04

**Authors:** Gustavo Pacheco-López, Marcel Pérez-Morales, Kioko Rubí Guzmán-Ramos, Johnny Davis Figueroa, Ute Krügel, Javier A. Bravo

**Affiliations:** ^1^Health Sciences Department, Metropolitan Autonomous University (UAM), Campus Lerma, Lerma, Mexico; ^2^School of Medicine, Loma Linda University, Loma Linda, CA, United States; ^3^Medical Faculty, Leipzig University, Leipzig, Germany; ^4^Pontificia Universidad Católica de Valparaiso, Valparaiso, Chile

**Keywords:** obesogenic environments, neurodevelopmental diseases, neurodegenerative diseases, obesity, energy homeostasis

The classical debate between nativism (nature, genes) and empiricism (environment, nurture) originated in ancient Greece and developed in the Renaissance, including analogies of Aristotle and metaphors of Gottfried Wilhelm Leibniz about the contents of the mind, persists to our days in neurobiology. The subject of this Research Topic is an example of this controversy. From a methodological perspective, an obesogenic environment is an experimental approach in which environmental manipulations, such as exposure to hypercaloric diets, drinking sugar-sweetened beverages (i.e., diet-induced obesity), and reduced physical activity, separately or in combination, may lead to metabolic imbalances, weight gain, and finally to adiposity. The scientific field continues making strides to determine the contribution of the myriad of obesogenic factors modulating energy homeostasis.

In this Research Topic, six original papers, two reviews, and one mini-review summarize the impact of several obesogenic factors upon food intake regulation, obesity-associated biomarkers, and describe several behavioral and molecular approaches that enhance our understanding of obesity. In the context of the sedentary lifestyle and the constant availability of junk food that has dramatically increased in the last 30 years all over the world, Flores-Dorantes et al. provide an integrative view on the relationship between obesogenic environments and obesity, with both neurodegenerative and neurodevelopmental diseases. The authors emphasize the involvement of genes traditionally associated with food intake regulation, such as those that code for the leptin-melanocortin pathway proteins and their incidence on neurodegenerative and neurodevelopmental diseases. They call our attention to the gene-environment interaction that promotes an imbalance between caloric intake and energy expenditure. They provide further evidence for common neurobiological pathways between obesity and neurodegenerative diseases, suggesting that metabolic syndrome and obesity are risk factors for Parkinson's disease and Alzheimer's disease (AD).

Whereas a relationship between obesity and the development of neurodegenerative diseases is widely accepted, we are just beginning to understand how environmental factors and the microbiome contribute to these conditions. Bello-Medina et al. demonstrate evidence for spatial memory deficits, differences in beta diversity, and lower relative abundances of *Actinobacteria* and *TM7* in the gut microbiota of 3 months-old 3xTg-AD mice, one of the most used transgenic models of AD, compared to non-transgenic mice. Their results suggest that microbiota alterations could be related causally to cognitive decline and may be employed as non-invasive biomarkers of AD, even at a presymptomatic stage.

Peleg-Raibstein focuses on the impact of maternal overnutrition by highly palatable diets, high-fat diets, a combination of high-fat-high-sugar diets, or cafeteria diets on the development of obesity and cardio-metabolic dysfunction and cognitive aging in the progeny. The article emphasizes the risk of unhealthy food environments provided by parents and the consequent exposure of their children. The author points to difficulties to discern between genetic and environmental risk factors to develop obesity and brain pathologies in the offspring. Related to these concepts, Cruz-Carrillo et al. employed a 9-week maternal overnutrition protocol. The authors report that the offspring from cafeteria diet-fed mothers exhibit an increased motivation to obtain pellets in operant tasks and reinforce their orexigenic response following subcutaneous injection of ghrelin. This behavior was associated with a higher global methylation in the shell region of the nucleus accumbens (NAcSh) and correlated with changes in the expression of pro-inflammatory cytokines. These results propose that high caloric nutritional intake during pregnancy and lactation increases the susceptibility to develop addictive-like behavior patterns in the progeny.

Apart from influencing obesity and metabolic dysfunction, Tsan et al. review literature in which overconsumption of highly palatable and dense foods leads to long-lasting multiple neurocognitive impairments in diverse rodent models. They conclude that overconsumption of *Western diets* during critical neurodevelopment stages is associated with detrimental cognitive performance across the lifespan. An example of this intricated relationship is given by Vega-Torres et al. In their study, the consumption of a *Western-like* high-saturated diet by adolescent rats for only 1 week is sufficient to impair emotional reactivity and anxiety. Cued fear extinction memory and neuronal activation of the basomedial amygdala attenuated, and corticosterone mRNA levels in the medial prefrontal cortex increased, altogether reveal the harmful impact of short-term exposure to obesogenic environments on the corticolimbic circuitry and emotional regulation during specific stages of neurodevelopment. Another study provided by Han et al. investigated the effect of a much more chronic exposure of young C57BL/6J mice to a high-fat diet. In their study, body weight, anxiety-like behavior, and, at the molecular level, the midbrain content of dopamine and D2 dopamine receptors increased after chronic diet exposure. From the pharmacological perspective and in line with food intake-associated dopaminergic activity, Kalyanasundar et al. evaluated the effect of D-norpseudoephedrine (NPE, also Cathine), an amphetamine-like sympathomimetic and anorexigenic drug, on food intake, body weight loss, and locomotion. Their results suggest that NPE-induced anorexia and body weight loss modulates neuronal spiking activity in the NAcSh. Though NPE is an African lifestyle drug with addiction risk, as an experimental tool, it elucidates feeding regulation associated with NAcSh neurons' activity in part controlled by midbrain dopaminergic neurons. Regarding the genetic aspects of metabolic diseases, Amaya et al. performed a translational study in AdKO_2.0_ transgenic mice, an animal model of Cushing's syndrome (CS), which is characterized by hypercorticosteronemia and metabolic abnormalities predisposing for obesity and metabolic syndrome, due to an inactivation of the gene encoding the regulatory subunit 1 alpha of the PKA, targeted to the adrenal cortex. Long-term exposure to high plasma glucocorticoids is strongly related to the gain of visceral tissue and stimulation of lipogenic pathways triggered by chronic stress. Their study shows overall parallels between imaging data from CS patients and AdKO_2.0_ transgenic mice as there is volume reduction in several brain areas and lower astrocytic and microglial markers, providing the opportunity to study the molecular basis and consequences of hypercorticosteronemia in a preclinical model.

The articles included in this Research Topic contribute to a more profound view of the complex relationship and potential causality between various obesogenic environmental conditions with the etiology of relevant neurodevelopmental and neurodegenerative diseases. The editors of this topic want to thank all the authors for their important contributions.

## Author Contributions

All authors listed have made a substantial, direct and intellectual contribution to the work, and approved it for publication.

## Conflict of Interest

The authors declare that the research was conducted in the absence of any commercial or financial relationships that could be construed as a potential conflict of interest.

## Publisher's Note

All claims expressed in this article are solely those of the authors and do not necessarily represent those of their affiliated organizations, or those of the publisher, the editors and the reviewers. Any product that may be evaluated in this article, or claim that may be made by its manufacturer, is not guaranteed or endorsed by the publisher.

